# The Challenges of Postoperative Tissue Flap Vitality Monitoring in Obese Individuals

**DOI:** 10.3390/jcm14217777

**Published:** 2025-11-02

**Authors:** Jerzy Jankau, Ignacy Rogoń, Mariusz Kaczmarek, Agnieszka Rogoń, Monika Stołyhwo-Gofron, Jerzy Wtorek

**Affiliations:** 1 Plastic Surgery Division, Gdańsk Medical University and University Hospitals, 80-210 Gdansk, Poland; jjankau@gumed.edu.pl; 2 Biomedical Engineering Department, Faculty of Electronics, Telecommunication and Informatics, Gdansk University of Technology, 80-233 Gdansk, Poland; mariusz.kaczmarek@pg.edu.pl (M.K.); jerwtore@pg.edu.pl (J.W.); 3 St. Adalbert’s Hospital, 80-462 Gdansk, Poland; arogon@copernicus.gda.pl; 4 Mediart Obesity and Overweight Treatment Clinic, Złota Karczma 25, 80-298 Gdansk, Poland; stolyhwo-gofron@wp.pl

**Keywords:** obesity, diagnostics, adipose tissue, tissue blood supply, tissue properties, flap reconstructions, tissue vitality

## Abstract

The global rise in obesity presents significant challenges for reconstructive surgery. Effective postoperative monitoring of tissue flap vitality is essential for successful outcomes, but obesity introduces complexities that can hinder accurate assessments. This article examines the specific challenges associated with monitoring tissue flap viability in individuals with obesity, focusing on how obesity-related physiological changes affect the effectiveness of various monitoring techniques. We explore alterations in subcutaneous adipose tissue—such as thickness, density, and volume—as well as changes in vessel structure, blood pressure, glucose levels, lactate levels, and tissue perfusion. These factors can impact the accuracy and reliability of monitoring methods. A deep understanding of these challenges is crucial for determining the suitability of different monitoring systems for patients with obesity undergoing flap reconstruction. Based on a review of clinical experience, we assess the usability of widely used monitoring devices, including ultrasound Doppler, near-infrared spectroscopy, and laser Doppler flowmetry, in patients with obesity. Non-conventional techniques such as glucose and lactate measurements, thermography, and fluorescence angiography are also investigated. This article aims to provide a comprehensive overview of the relationship between obesity and tissue flap monitoring, ultimately helping to select the most appropriate and effective methods for this patient population. By considering these factors, surgeons in cooperation with engineers can optimize postoperative care and enhance the likelihood of successful flap reconstruction.

## 1. Introduction

The 2024 study published by *The Lancet* shows that, worldwide, obesity among adults has more than doubled since 1990, and has quadrupled among children and adolescents (5 to 19 years old). The data also show that 43% of adults were overweight in 2022. As of 2022, more than 1 billion people in the world are living with obesity [1]. From a technical standpoint, patients with obesity may encounter issues revolving around improper functioning of medical instrumentation. Individuals with obesity pose challenges to the effectiveness and safety of Magnetic Resonance Imaging [2]. The challenges of accommodating larger patients and achieving optimal image quality in Computed Tomography Imaging for individuals with obesity are acknowledged [3]. Surgeons often view obesity in patients as a surgical risk factor [4,5,6]. The authors of an article [7] discussed potential shortcomings that may exist in breast imaging and interventions for patients who are overweight or obese. The surgeon performing breast reconstruction cannot truly evaluate the procedure as successful until the flap has survived the first few days. Postoperative observations start in the operating room, immediately after the release of the microvascular clamps. Postoperative monitoring is irreplaceable in achieving a high rate of success in tissue transfer [8]. There is no method that is universally effective. In this article, we present and review usability of clinically acclaimed and experimental monitoring techniques suitable for flap vitality in patients with obesity.

The body mass index (BMI) is calculated as a person’s weight in kilograms divided by their height in meters squared. Obesity, an excess accumulation of body fat, is generally defined as a BMI of 30 kg/m^2^ and higher and overweight is defined as a BMI between 25 and 30 kg/m^2^ [9,10]. In Table 1, classification of overweight and obesity according to the National Heart, Lung, and Blood Institute (NHLBI) and World Health Organization (WHO) is presented. In young adults, normal levels of body fat are considered to be 12–20% body weight in males and 20–30% body weight in females, while levels of >25% body weight in males and >33% body weight in females can be considered obese [11]. The method for calculation of percentage body fat from the BMI is presented in Table 2.

In a study by Hanwright et al. [16], the researchers analyzed how the BMI affected breast reconstruction in nearly 13,000 patients from various medical institutions. They found that postoperative complications were significantly higher in patients with obesity undergoing all types of reconstruction. A meta-analysis conducted by Panayi and co-authors [17] revealed that women with obesity were 2.29 times more likely to experience surgical complications, 2.89 times more likely to have medical complications, and 1.91 times more likely to need additional surgery for both prosthetic and autologous reconstruction. Another large study led by Fischer and co-workers [18] showed that increased obesity was linked to higher rates of overall perioperative complications, longer hospital stays, longer operation times, and greater anesthesia risks. Specifically, class III patients with obesity had a 5.3% higher risk of returning to the operating room and a 1.7% higher rate of flap or implant loss within 30 days.

According the Lancet Commission report on the definition and diagnostic criteria of clinical obesity, adopting a more precise approach to identifying obesity is required and it should aim to improve the lives of individuals living with obesity [19]. Proposed by Sharma and Kushner [20], the Edmonton Obesity Staging System (EOSS) is a classification system designed to assess the health impact of obesity beyond just weight or body mass index (BMI). Unlike the BMI, which only measures body weight relative to height, the EOSS evaluates the presence and severity of obesity-related health conditions to provide a more accurate risk assessment. Clinical and Functional Staging of Obesity: Five stages of obesity are characterized in Table 3. The anthropometric classification of obesity based on the BMI is crucial in population studies. It highlights the significant increase in obesity rates and its correlation with morbidity and mortality. However, this measure has clear limitations when it comes to making clinical decisions for individuals. BMI is an uncertain diagnostic index of obesity [21]. Individuals with the same BMI may have a nearly twofold difference in total body fat. Conversely, individuals with identical total body fat can exhibit a wide range of BMI values [22]. Relying exclusively on the BMI for making clinical decisions in obesity treatment is insufficient due to its inability to personalize health status stratification [23,24]. Researchers have observed an independent and complementary role of the BMI and EOSS as predictors [25]. EOSS was significantly better than the BMI for predicting polypharmacy and health service use [26]. Additionally, EOSS can be also used for risk stratification in the clinical setting. According to the large German cohort study [27], patients with EOSS ≥ 3 showed the highest postoperative complications and the highest mortality.

## 2. Most Common Postoperative Complications in Patients with Obesity

In Table 4, articles by Knox et al. [28], Seidenstuecker et al. [29], Ochoa et al. [30], Wilkins et al. [31], Fischer et al. [32], Palve et al. [33], Heidekrueger et al. [34], and Yoshino et al. [35] in which authors have shown the correlation between patients’ BMI and complications in autologous breast reconstruction surgeries were presented. Researchers agree that an elevated BMI value should be considered a risk factor for a higher risk of complications when performing natural breast reconstruction. Across studies, a higher BMI consistently emerged as a risk factor for complications in flap surgeries, with significant differences between BMI groups. Surgery types, especially DIEP and pTRAM, showed varying rates of complications depending on the BMI, with obesity strongly linked to outcomes like seroma, flap failure, and delayed healing. Moreover, the existence of numerous premises supporting this thesis justifies inspecting whether it is worth considering the value of the BMI and other demographics such as age, smoking record, and history of chemotherapy assessed while determining the time of post-surgical monitoring. It may even be useful to create an algorithm of surveillance time based on those factors in the future. Researchers point out that no significant difference regarding the rate of partial and total flap loss between all BMI groups can be noticed but the complications may appear more commonly in those with a BMI >25. On the other hand, an observation made by Kim et al. in 2013 allows us to make an assumption that underweight patients should also be cautiously observed in the post-surgical period [36]. It is also worth noticing that based on some reports, the BMI is not the best tool to predict post-surgical complications, since it fails to accurately reflect the distribution of abdominal adipose tissue. Measuring the thickness of the abdominal wall by performing a CT scan should be considered the better prognostic method [37]. While autologous abdominally based free flap reconstruction can be successfully performed on patients with various body types, obesity is linked to significantly higher rates of complications in both the breast and abdomen. In the most recent study [38], researchers indicated that the optimal body mass index (BMI) cutoffs for minimizing complications are as follows: a BMI of 32.7 kg/m^2^; for breast complications and a BMI of 30.0 kg/m^2^ for abdominal complications. Furthermore, having a BMI greater than 32.7 kg/m^2^ is as predictive of breast complications as current smoking status, while a BMI greater than 30.0 kg/m^2^ is a stronger predictor than age, smoking, or diabetes when it comes to abdominal complications. Authors of another study suggest that the risks of postoperative complications following free flap breast reconstruction are highest for patients with a BMI greater than or equal to 35 kg/m^2^, having a nearly 1.5 times higher likelihood of postoperative complications [39]. Interestingly, another 2024 study indicates that microsurgical breast reconstruction can be performed safely and efficiently in patients with obesity [40].

## 3. Obesity-Induced Changes in Human Body Composition and the Selected Properties

Obesity induces significant alterations in human body composition and various physiological properties, impacting overall health. Key changes include adipose tissue remodeling [41], skeletal muscle functioning, metabolic and cardiovascular implications [42], and alteration of bone marrow homeostasis [43]. In this section, only selected aspects of obesity-induced changes are discussed. The selection was performed based on essential properties from a considered monitoring techniques perspective.

### 3.1. Subcutaneous Adipose Tissue Density and Volume

The tissue of a patient with obesity is characterized by increased subcutaneous fat, larger skin folds, and higher surface roughness [44]. The thickness of the skin is not affected by obesity. In general, higher adipose tissue thickness is observed in patients with a higher BMI. In the study of J. Choo et al. [45], the correlation between BMI and flap thickness was shown. According to the researchers findings, a 1-point increase in BMI led to a 1.11 mm increase in Latumus Dorsi flap thickness and an increase in the thickness of the subfascial fat layer by 0.513 mm. The thickness of the subfascial layer closely correlated with the BMI. The contribution of the subfascial layer to the overall flap thickness tends to increase as a percentage of the overall flap thickness with an increasing BMI. Authors of another study [46] examined how BMI affects flap thickness, mainly focusing on the subfascial and superficial fat layers in anterolateral thigh (ALT) flaps. The thickness of both male and female flaps had a positive correlation with the BMI and the strongest correlation was found for subfascial ALT thickness in female patients. Breast tissue coverage refers to the tissue located between the skin and the Cooper ligaments that surround the gland, specifically the dermis and subcutaneous fat. According to the study by Rancati et al. [47], the breast tissue coverage thickness in a sample of 176 Caucasian women ranged from 0.233 to 4.423 cm, with a mean value of 1.952 cm. A comparison of tissue coverage and breast volume revealed a non-direct relationship between these factors.

Obesity per se has not been described to affect the density of adipose tissue. It is the quality of the fatty tissue that can cause changes in its density. The low density of subcutaneous and visceral adipose tissue is associated with adverse levels of circulating adipokines and is rather independent of generalized adiposity (BMI), central obesity (waist circumference), or respective abdominal adipose tissue quantity [48]. Lower attenuation of the adipose tissue (Hounsfield Units, HU) was associated with larger adipocyte size, as well as lower adiponectin and higher leptin levels [49]. Furthermore, findings in the article indicated the influence of the BMI on the density of adipose tissue. Individuals with the lowest body mass index had the highest density of adipose tissue. Conversely, those with the highest body mass index exhibited the lowest adipose tissue density. This data is supported by another study. In [50], authors indicate that as the BMI increases, the density of adipose tissue tends to decrease. This is primarily due to the accumulation of lipids within adipocytes, leading to larger cell sizes and reduced tissue density.

### 3.2. Changes in Vessel Structure and Properties

Almost all blood vessels are surrounded by perivascular adipose tissue (PVAT), which regulates vascular function. This is a specialized local deposit of adipose tissue surrounding blood vessels that also provides mechanical protection and regulation of blood vessel tone [51]. In obesity, PVAT becomes dysfunctional and exerts detrimental effects on the blood vessels [52]. It becomes highly inflamed and induces vascular dysfunction by the secretion of vasoconstriction factors and pro-inflammatory adipokines, the latter of which are important contributors to endothelial activation, vascular inflammation, and neointimal formation [53]. An increased amount of fatty tissue in obesity promotes the production of molecules such as resistin and chemerin, which result in atherosclerosis [54]. Other changes in adipose tissue in individuals with obesity may be overall defined as “ominous triad”, consisting of inflammation, fibrosis, and impaired angiogenesis [55]. Studies also suggest that the vascular wall of people with obesity is more permeable [56].

In the study, consisting of 66 cases, Scott et al. [57] did not find differences between age or BMI and the number of perforators or average perforator size per patient. However, a significant positive linear association was found between the average perforator diameter and the total number of them in the area of the abdominal wall. Authors concluded that there is no anatomical difference in perforator quality among patients with a BMI under 35. According to Shayan et al. [58], body weight gain causes irreversible dilatation of the cutaneous perforators of the abdominal wall. Moreover subsequent body weight loss does not decrease the perforators’ size and does not result in facilitating optimal flap harvest in perforator flap surgery. Yet, the preoperative weight loss benefits both flap harvest and operative outcomes. Similar results have been presented by Sacher et al. [59], who indicate the correlation of BMI and abdominal wall thickness with the diameter of the dominant deep inferior epigastric artery perforator (DIEP).

In the skin tissue, larger perforators (>0.5 mm in diameter) were found to have a direct course through the subcutaneous fat to the skin. Smaller perforators do not reach the skin but terminate at the level of the deep fat layer by branching after piercing the rectus sheath. The direct perforator vessels with their associated veins (microdissection) keep a consistent diameter before dividing at the subdermal level and end by contributing to the subdermal plexus [60].

### 3.3. Blood Pressure Changes

Obesity is highly correlated with the risk of hypertension. The relationship between excess adiposity and increased blood pressure is well established, and it is estimated that obesity accounts for 65–78 percent of cases of primary hypertension. The mechanisms through which obesity causes hypertension are complex and include sympathetic nervous system overactivation, stimulation of the renin–angiotensin–aldosterone system, alterations in adipose-derived cytokines, insulin resistance, and structural and functional renal changes [61].

In the study performed in 2021 in Bangladesh, over 1300 individuals were examined to estimate the relation between hypertension and obesity. The results showed that overweight adults had a 61 percent higher risk of hypertension, while the risk was 2.35 times higher for individuals with obesity, compared to those with normal weight [62]. A study by Yusni et al. indicated associations between body weight and blood pressure. According to the findings, there is a strong significant correlation between body weight and systolic blood pressure, whereas the correlation between body weight and diastolic blood pressure is moderate. A positive association between body weight, BMI, and blood pressure was proved. An increase in body weight of 1 kg results in an increase in systolic blood pressure (SBP) of 0.725 mmHg and diastolic blood pressure (DBP) of 0.318 mmHg. In addition, an increase in the BMI of 1 kg/m^2^ is followed by an increase in SBP and DBP of 1.6 mmHg and 0.834 mmHg, respectively [63]. The changes, however, can be reversed by weight loss. A reduction of 1 kg of weight has been associated with SBP and DBP reduction of 0.3–0.5 mmHg/kg. Results applied to both healthy and hypertension-affected individuals [64]. A study on Italian children with obesity found that their cardiovascular autonomic regulation had an altered profile compared to normal-weight children. Children with obesity exhibited higher SBP and an increased low-frequency variability power of SBP, which indicates enhanced vasomotor sympathetic regulation. Additionally, children with obesity showed a lower spontaneous baroreflex gain. Notably, both groups had similar values for heart rate and heart rate variability [65].

### 3.4. Blood and Tissue Glucose Levels

While considering challenges in postoperative tissue flap vitality monitoring in individuals with obesity, it is essential to mention diabetes mellitus (DM). It is one of the most common complications of obesity. Obesity or excessive weight gain is identified as the most important and significant risk factor in the development and progression of type II DM [66]. It has been proven that the first class of obesity is associated with a five times increased risk of type II DM, which increases to a 12 times higher risk in those with class III. One mechanism linking obesity to type II DM is related to an increase in liver and pancreatic visceral fat. Excess hepatic triglycerides are transported in very-low-density lipoproteins to all tissues, including the beta-cells of the pancreas, and over many years, this results in progressive pancreatic beta-cell dedifferentiation with a subsequent relatively sudden onset of clinical diabetes [67]. DM has been associated in many studies with a higher risk of postoperative complications such as infections, wound healing disorders, hematoma or reoperation, postoperative hospital readmission, or even death [68].

Xiaohan Liu et al. [69] have observed that in a group of over 1900 patients with diabetes, both types 1 and 2, the average glucose reading in the right arm was higher 96% of the time and was half as likely to detect glucose levels outside the 70–140 mg/dL range compared to the left arm. When using a subcutaneous device for continuous glucose monitoring, Wheizao Lu and his team have created an “Atlas of glucose uptake across the entire human body as measured by the total-body PET/CT scanner” [70]. In their study, 18F-fluorodeoxyglucose was used to measure glucose uptake among 15 individuals. One of their discoveries was that the lateralized differences in mean standardized uptake value normalized by lean body mass existed in the kidney, while no lateralized differences were found in other organs across the body. Another report made by this team is that the glucose uptake may differ in major organs and body parts of healthy-weight and overweight groups. Overweight individuals demonstrated increased glucose uptake in the bilateral lungs and decreased glucose uptake in the brain, heart, stomach, and pancreas [71]. A. Kissebah et al. underline that women with upper body obesity have higher plasma glucose and insulin levels compared to those with lower body obesity [72]. This finding suggests that fat distribution plays a significant role in glucose metabolism, with upper body fat being more closely associated with severe metabolic complications.

Insulin-stimulated glucose uptake is higher in visceral than in subcutaneous adipose tissue. However, in individuals with obesity, both types of adipose tissue show lower glucose uptake, reflecting widespread insulin resistance [73]. Fasting serum glucose significantly increases in individuals with obesity [74]. This is due to poor glucose regulation associated with weight gain. One of the mechanisms explaining that fact is the development of insulin resistance in patients who are overweight. Excessive energy intake results in cell overload that triggers mechanisms to protect cells from further energy accumulation by reducing insulin sensitivity. Additionally, hypertrophied adipocytes and macrophage infiltration cause local inflammation that may result in general inflammation that induces insulin resistance [75]. Its metabolic consequences include, among others, hyperglycemia [76].

### 3.5. Lactate Levels and Acidosis

Lactate metabolism is also altered in obesity. Increasing obesity is associated with increased blood lactate levels [77]. Disorders of fat metabolism, decreased arterial flow to white adipose tissue, and a continuous low-grade inflammatory state secondary to hypoxia appear to be the triggers for metabolic changes such as increased resistance to insulin action, hyperglycemia, and hyperlactatemia [78,79].

In 2022, a study summing up 20 years of data was presented by Lambert et al. The authors concluded that metabolic diseases, including obesity, are characterized by metabolic acidosis and anion gap elevations in the absence of chronic kidney disease. Lactic acidosis is a type of anion gap metabolic acidosis. In that study, the authors used the term metabolic acidosis defined as serum bicarbonate ≤23 mEq/L and an elevated anion gap for the scores ≥95th percentile for the total population. The authors indicate that the exact source of this abnormality in obesity is uncertain and they suggest that it is not the higher BMI but the presence of pathologic fat mass associated with greater body mass, or metabolic dysfunction related to obesity, that mediates this disturbance [80].

Lactate levels are mildly but chronically elevated in obesity. Impaired oxidative capacity is also seen in these disease states in skeletal muscle, liver, and adipose tissue. Although the lactate anion is a base equivalent, its formation in response to energy demand is accompanied by metabolic acidosis if oxidative capacity is insufficient. For acid–base homeostasis to prevail, oxidative capacity must improve, and organic acid must be cleared primarily by the liver [81]. When the natural compensation mechanisms of the body are deficient, and the buffering capacity is exceeded, lactic acidosis may occur. The definition of lactic acidosis includes pH less than or equal to 7.35 and lactatemia greater than 2 mmol/L with a partial pressure of carbon dioxide (PaCO_2_) less than or equal to 42 mmHg. Normal lactate levels are less than 2 mmol/L, with hyperlactatemia defined as lactate levels between 2 mmol/L and 4 mmol/L. Severe levels of lactate are 4 mmol/L or higher [82].

DiNicolantino and O’Keefe postulate that the terms “metabolic acidosis” and “acidemia” should be distinguished. Acidemia, or too much acid in the blood, only occurs when the body’s buffering capacity can no longer maintain a normal pH level (in a range of 7.35–7.45). However, even at a normal blood pH, metabolic acidosis can occur. When the blood pH falls below 7.4, there is usually acid retention in the body and low-grade metabolic acidosis. However, the blood pH does not drop below the normal range until metabolic acidosis becomes severe. The authors suggest that a low blood pH is typically one of the last surrogate markers to become abnormal in those with low-grade metabolic acidosis and that it comes as an effect of years or decades of a state of low-grade metabolic acidosis due to acid retention and depletion in the bicarbonate reserves of the body. Metabolic acidosis primarily occurs inside the cell and in the interstitial fluid (fluid that surrounds tissues). The authors state that metabolic acidosis is a treatable condition with substantial adverse effects on human health, and its early recognition should be implemented [83].

It is not just obesity that can cause an increase in lactate levels and the acidic environment. A slight risk of developing severe lactic acidosis is sometimes linked with one of the treatments for diabetes. As mentioned before, DM II and obesity are often co-occurring. Metformin is a drug recommended as a first-line treatment in diabetes mellitus type II [84]. The efficacy of Metformin has been proved many times over the years. It has been shown to improve glycemic control through several mechanisms, the main one being the inhibition of hepatic gluconeogenesis. Moreover, Metformin is associated with many positive effects, such as having an impact on the prevention of microvascular and macrovascular complications. It is also thought to have beneficial effects that are not only the consequence of improved glucose control [85,86]. Overall, Metformin is considered a good and safe medication for DM type II [87]. However, in individuals with chronic kidney disease, it should be dosed carefully and should be skipped if the patient suffers from diseases that predispose them to the development of acute kidney injury. It is due to a small but real risk of lactic acidosis [88,89].

### 3.6. Tissue Perfusion

Tissue perfusion is strictly linked to patency in perforators. If the blood flow in the perforator is compromised, the chances of the flap’s survival decrease. Flap blood perfusion according to Woodcock [90] depends on flap thickness and is in a range from 25 to 120 [mL/(min kg)]. In the study by Lorenzetti et al., authors evaluated the hemodynamics in different types of free microvascular flaps by measuring intraoperative transit-time flow [91]. Blood flow in the donor artery was greatest in radial artery flaps (averaging 57.5 mL/min), but this flow decreased dramatically (to about 6.1 mL/min) after the artery was connected to the flap. Conversely, the recipient artery in TRAM flaps initially had the lowest flow (around 4.9 mL/min), but flow increased significantly (to approximately 13.7 mL/min) after connection, reaching a similar level to the donor artery. In muscle flaps, donor artery flow averaged 15.9 mL/min and increased to 23.9 mL/min after anastomosis. The radial forearm flap exhibited the highest blood flow in relation to tissue weight. In one failed muscle flap, blood flow in the pedicle after anastomosing was the lowest found (3.6 mL/min) in muscle flaps. It cannot be said that such a low value would be predictive of free flap failure (the same value was also found in three successful radial forearm flaps), but it might be predictive of a critical hemodynamic condition that can cause thrombosis, especially in muscle flaps which seem to have a more variable flow (range of 6 to 47 mL/min) than the radial forearm flap (range of 3.4 to 9 mL/min) or the TRAM flap (range of 6 to 23 mL/min). An association between obesity and changes in the perfusion of adipose tissue blood flow was observed. Typically, an overnight fast abdominal adipose tissue blood flow (ATBF) is 3–5 mL/100 g of tissue/min. In obese and/or insulin-resistant individuals, fasting ATBF is lower than in healthy normal-weight individuals and its responsiveness to nutrients is reduced in individuals with obesity. Other factors that could affect ATBF such as location and sex were examined. No sex-specific differences have been found in basal ATBF. It seems to be higher in the abdominal region than in femoral adipose tissue. Nevertheless, a rather large intra-individual variation in ATBF has been recognized [92].

Skin vasculature is also affected by changes due to obesity. The major determinants of dermal blood content are capillary density (the number of capillaries per area of tissue) and cutaneous blood flow (the volume of blood per unit time). Decreased dermal capillary density with increasing obesity has been demonstrated in various studies performed on different populations [93]. Moreover, the team of Francischetti et al. presented a study in which they discovered that individuals with obesity affected with metabolic syndrome lack functional capillary reserve [94]. This may be related to insulin resistance. Patients with obesity have structural and functional alterations in skin microcirculation that are proportional to the increase in the degree of global and central obesity. The outcomes of studies analyzing the effect of obesity on the skin blood flow vary. The team of Mori et al. presented a conclusion that the cutaneous blood flow increases as the BMI increases [95]. On the other hand, when Kılıç et al. examined microcirculation of individuals with and without obesity, the blood flow was significantly higher in the second group, both in the resting state and after performing a post-occlusive reactive hyperemia test [96].

Metabolic dysfunction-associated steatotic liver disease (MASLD) is a disease associated with visceral obesity, and its serious consequences include the occurrence of components of metabolic syndrome [97,98]. The relationship between MASLD and obesity is linear [99]. Patients with MASLD are more likely to develop cardiovascular diseases than those with normal liver function, especially cardiovascular diseases associated with atherosclerosis [100]. The development of MASLD is mainly caused by an impaired function of visceral adipose tissue (VAT), promoting the release of free acid fats, hormones, and adipokines. It should be noted that an increase in the deposit of both visceral and subcutaneous adipose tissue by 1% causes an increase in the accumulation of lipids in the liver by 40% and 20%, respectively [101]. In overweight and obesity, adipocytes undergo hyperplasia or hypertrophy due to an excessive supply and storage of nutrients. The hypertrophy process leads to tissue hypoxia and induces a number of unfavorable activation processes; as a consequence of which insulin resistance develops, the secretion of adipokines (i.e., adiponectin and leptin) changes and the function of mitochondria is impaired [102].

### 3.7. Temperature Distribution

Based on the analysis of the literature reports, the influence of adipose tissue on the visible temperature on the skin surface is linked to lower conductivity. Adipose tissue’s conductivity (typically cited around 0.16–0.20 W/m·K) is lower than lean tissue such as muscle (approximately 0.40–0.50 W/m·K) [103,104]. As a result, individuals with obesity generally have lower average thermal conductivity across the skin and subcutaneous layer. This lower conductivity decreases heat transfer from the core to the periphery, meaning obese skin often has a lower surface temperature under resting conditions. Another important parameter is thermal capacity, or more precisely, specific heat capacity, which is the amount of heat required to raise the temperature of 1 kg of a substance by 1 °C (or 1 K). Different tissues (e.g., fat and muscle) have varying water contents and compositions, leading to differences in their specific heat capacities. Fat cells also contain lipids, which generally have a lower capacity to store heat than water-rich tissues. Although adipose tissue’s lower thermal capacity might suggest that it can warm or cool faster, its low thermal conductivity slows heat transfer into or out of the tissue [104,105]. The typical value for adipose (fat) tissue has a lower specific heat capacity than muscle tissue. The literature data suggest values around 2.0–2.3 kJ/kg·K for human adipose tissue [103,104]. Lean (muscle) tissue has a higher water content; thus, it typically exhibits a higher specific heat capacity, often reported in the 3.0–3.4 kJ/kg·K range [103,105]. In conclusion, attention should be paid to the following mechanisms observed in people with obesity: Thermal Insulation: Adipose tissue is a poor conductor of heat, forming a natural insulating barrier [105]. As a result, regions with thick subcutaneous fat layers often show lower baseline skin temperatures at rest [104]. Altered Blood Flow Dynamics: Obesity is frequently associated with endothelial dysfunction and changes in microcirculation [105,106]. Reduced blood flow impairs heat transfer to the skin, contributing to cooler surface temperatures. After a thermal challenge (cold or heat), the time to return to baseline is often prolonged in individuals with obesity. Regional Differences: Abdomen and Thighs—Typically higher subcutaneous fat thickness, which leads to cooler and more uniform temperature distributions. Hands and Feet—Can be cooler at rest but may exhibit larger relative changes during thermal challenges if vasodilation is triggered [103,104,105].

## 4. Monitoring Challenges

Postoperative monitoring significantly contributes to the high success rate of tissue transfer [8]. While the literature presents various techniques, a universally effective method is yet to be established. The ideal flap monitoring technique should be reliable, reproducible, easy to interpret, non-invasive, safe, inexpensive, and user-friendly. Moreover, the perfect method should accurately distinguish between vessel spasm and occlusion, as well as identify venous complications from arterial ones. Increased rates of complications in patients with obesity who undergo autologous breast reconstruction are well characterized in the literature [17,107,108,109,110]. Cases of elevated rates of delayed wound healing of the donor site, total flap loss, and mastectomy skin-flap necrosis are widely reported, including elevated rates of delayed wound healing of the donor site, total flap loss, and mastectomy skin-flap necrosis. It is important to note that elderly patients tend to experience an increased rate of complications and longer hospitalization due to comorbidities [111]. According to the authors of a large study [112], female sex, peripheral vascular disease, and preoperative thrombocytosis are predictive of early complete free flap failure. This suggests that a hypercoagulable state and poor vessel quality may predispose patients to flap failure.

### 4.1. Type of Monitoring Procedure

**Figure 1:**
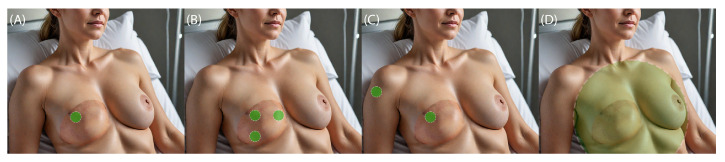
Types of monitoring procedures: (**A**) Point, (**B**) multi-point, (**C**) differential, and (**D**) total flap. Green dots indicate monitored area.

Point measurement techniques are the least complex. They provide a quick assessment of the tissue state. However, only a small area of the tissue is observed. Thus, results may not accurately reflect the overall health of the entire flap. Furthermore, point measurements may not be sensitive enough to detect subtle changes. Such downfalls are mitigated by a multi-point sensor setup (Figure 1). With a differential setup, sensitivity is higher due to a reduction in noise and an increase in resolution. Also, the baseline shifting phenomenon should be considered. An increased number of measurement points forces more intricate construction of the device and introduces an issue of proper calibration of the sensors. It is important that the influence of the sensors on themselves is accounted for. A specific case of multi-point measurement is differential measurement, where one of two identical sensors is placed on the tissue of interest, while the other serves as a reference. This approach helps to eliminate common mode errors such as a change in lightning or metabolic spikes that could lead to misinterpretation of the results. Total flap monitoring techniques require the employment of cameras and processing software. Among the pros of such techniques, the ability to observe changes in the whole flap is the most important. It allows us not only to trace the changes but also their origin. On the other hand, patient monitoring with the use of a camera may cause certain discomfort, as the flap has to be exposed throughout the whole procedure.

### 4.2. Velocity vs. Volumetric Measurements

Both velocity and volumetric measurements provide valuable information about flap perfusion. Pressure pulses cause arteries and arterioles to distend (expand) and recoil (contract). By observing changes in the volume of the vessels, volumetric measurements can estimate the amount of blood flowing through the vessels at a given time. Photopletysmography is a widely used, non-invasive optical technique for measuring blood volume changes in the microvascular tissue. It operates by detecting variations in light absorption due to pulsatile blood flow, typically using infrared or visible light. Volumetric measurements can be obtained with a consumer-grade digital camera applying video magnification techniques such as Eulerian Video Magnification (EVM) and motion-based magnification [113,114]. It is important to note that even when the vessel is blocked by plaque or a clot, the pressure wave from the heart can still travel through the artery, causing blood to pulsate. However, very little blood, if any, actually goes past the blockage to reach the tissues. A strong pulse does not always guarantee adequate perfusion of the tissues, particularly in cases where there is vascular obstruction or stenosis downstream. In this scenario, measurements of blood flow velocity are useful. Instead of observing the volume changes caused by blood flow, we observe the actual flow of blood through the vessels, delivering oxygen and nutrients to tissues and removing waste products. Tissue perfusion can be obtained with the use of an ultrasound Doppler. An ultrasound transducer emits high-frequency sound waves that travel through the body and bounce off moving red blood cells, causing a frequency shift. The frequency of the reflected sound waves changes depending on the speed and direction of blood flow [115]. Electromagnetic flowmetry using a magnetic field to measure the Pulse Wave Velocity [116] can also be employed.

### 4.3. Monitoring Period and Time Window

No specific recommendations for the monitoring of patients with obesity were found in the literature. Most flap failures occur within the first 48 h [117]. Some studies suggest that all microvascular complications can be detected within the first 24 h, supporting the idea that intensive monitoring beyond this period may not be necessary in certain cases [118]. Some studies suggest a reduction to 15 h, without increasing the risk of missing flap failures [119]. Although the probability of complications is lower compared with the first 48 h, some pedicle thrombosis cases and most hematomas requiring reoperation may occur within 72 h.

The authors of the study [120] suggest that the close monitoring of free flaps is most critical during the first 24 to 48 h, when most thrombotic complications occur, and that prompt identification and re-exploration is critical. In the article [121], the authors underline that the postoperative perfusion characteristics of the DIEP flap showed a critical drop in the cutaneous oxygen supply and blood flow on the third postoperative day. This represents a potential risk and should be considered in the postoperative management of the DIEP flap. The authors of studies on rodents underline that early detection and timely intervention within this window significantly improve the likelihood of successful flap salvage [122]. The critical time window for salvaging a compromised flap after a vascular compromise is within the first 6 hours. While flaps can sometimes be salvaged after 12–24 h of ischemia, the success rate diminishes, particularly as ischemic tolerance reduces after prolonged ischemia. Studies indicate that interventions beyond the 6 h mark still offer some salvage potential, but the success rate is significantly lower due to prolonged ischemia-induced damage [123]. Moreover, the secondary critical ischemia time shows a significant decrease in flap survival as ischemia duration increases, indicating that timely intervention is crucial for both initial and secondary ischemia events [124].

Authors of the paper [121] suggest that monitoring should be performed every 1–2 h to ensure the timely detection of potential issues and prompt intervention. According to the guidelines for Free Flap Monitoring by NHSGGC Paediatrics, monitoring intervals of 30 min are recommended [125]. A study by Hirigoyen et al. [126] revealed that 75% of surgeons preferred to perform monitoring at intervals of three hours or less, 23% opted for intervals between 3 to 6 h, and only 2% were comfortable with a monitoring gap of more than six hours. After the initial 48 h period, if the flap is stable, the frequency of monitoring can be reduced to every 4–6 h, with regular clinical assessments continuing [127]. Another study shows that monitoring protocols can vary widely—from simple 2 h intervals for 3 days to more complex ones every 4 h for 2 days and every 8 h for 1 week [128]. In the study involving mobile photoplethysmograph (PPG), researchers proposed the following protocol: 3 h for the first 24 h, every 4 h the second day, every 6 h the third day, and twice daily until discharge [125]. Currently in our clinic, monitoring is conducted every 2 h for the first 12 h, then every 4 h for the next 12 h, and subsequently, every 6 h for 24 h. After this period, we discontinue further monitoring unless there is a specific reason to continue. According to Siemionow and Arslan, free flap salvage rates due to compromised perfusion are inversely proportional to the time interval between the onset of ischemia and the surgical operation [129].

## 5. Monitoring Techniques

In the most recent review [130], clinically acclaimed and experimental flap monitoring techniques were presented. Based on the gathered data and our clinical experience, the most promising devices for monitoring individuals who are overweight or obese were chosen. The following section presents a description of the construction and technical analysis of such methods. Monitoring techniques were categorized into direct methods, which observe blood flow, and indirect methods, which examine the effects of impaired blood flow.

### 5.1. Direct Techniques

#### 5.1.1. Handheld Doppler Ultrasonography

Handheld ultrasound Doppler devices are widely used in tissue vitality monitoring to assess blood flow and vascular health [131]. These portable systems utilize the Doppler effect to measure the movement of blood through vessels, allowing clinicians to evaluate tissue perfusion and detect potential problems such as ischemia, vascular occlusion, or poor tissue oxygenation.

The Doppler effect occurs when ultrasound waves emitted by the transducer interact with moving red blood cells in the bloodstream. The frequency of the reflected ultrasound waves shifts depending on the velocity and direction of blood flow [132]. The key component of a Doppler ultrasound device is the transducer, which emits and receives ultrasound waves. The transducer is typically compact, enabling handheld operation. It generates high-frequency sound waves that penetrate tissues and interact with moving blood cells. Lower frequencies are used for deeper vessels and tissues but with lower resolution.

Obesity can pose challenges for Doppler ultrasonography due to the increased amount of subcutaneous fat tissue, which can attenuate the ultrasound signal. Lower frequency transducers have better penetration through adipose tissue, allowing for deeper imaging and detection of blood flow in deeper vessels [133]. Flow in larger vessels like arterioles and small arteries is detectable for ultrasound frequencies higher than 4 MHz [134]. However this frequency range remains strained to detect blood flow in vessels smaller than arterioles [135]. For the observation, superficial blood flow in small-vessel ultra-fast ultrasound imaging is employed [136,137]. The penetration depth of ultrasound is directly related to its wavelength. A general rule of thumb is that effective scanning can reach a depth of approximately 500 times the emitted wavelength within tissue. For a frequency of 7 MHz, this results in a penetration depth limit of about 11 cm [138].

Some handheld Doppler devices use continuous-wave transducers, emitting and receiving ultrasound signals continuously. This allows for the detection of high-velocity blood flow, ideal for superficial vessels. Such devices are characterized by high sensitivity and larger sample volume [139]. Handheld Doppler devices can also use pulsed-wave transducers, which emit ultrasound pulses at specific intervals. This method provides better depth resolution, making it suitable for localized tissue monitoring. Handheld Doppler devices may produce audible signals, where the pitch and intensity represent blood flow velocity, or visual waveforms displayed on an integrated screen or mobile device. Visual outputs typically include spectral waveforms showing blood flow over time. The consensus statement endorsed by the Society for Vascular Medicine and the Society for Vascular Ultrasound proposes a standardized nomenclature for arterial and venous spectral Doppler waveforms [140]. A high-frequency pulsed-wave Doppler transducer allows the interrogation of small microvessels, with a capability for estimating flow direction and absolute velocity [141]. This makes it invaluable for detecting early signs of vascular compromise or ischemia.

Handheld Doppler devices are handy for point-of-care monitoring in various settings. Its biggest advantages are general ease of use and immediate indication of flow impairment. Method pitfalls are linked to angle dependency [142] and the transducer’s operational frequency [143]. The difficulty of detecting low-flow or small-vessel perfusion is connected to high sensitivity and resulting motion artifacts [141]. Operator variability is also an important factor to consider. As the Doppler ultrasound is a non-continuous method, it forces the use of a measurement protocol with a specific time window. Commonly used simple acoustic Doppler devices provide only qualitative data, lacking the detailed anatomical or quantitative flow information that more advanced imaging systems offer.

Large and advanced ultrasound units enable the computed processing of acquired signals [144]. Color Doppler images are representations of Doppler shifts and commonly produced by a technique called auto-correlation. Its ability to detect small vessels and highlight suspicious areas is shadowed by low sensitivity and a reduced ability to show low velocity flow. Power Doppler (PD) works on a similar basis as Color Doppler; however, the processing method uses the integral of the power spectrum rather than estimating variance and mean frequency through auto-correlation. PD is very sensitive to low-level blood flow and it is not subject to aliasing. It being angle-independent is another highlight. On the other hand, PD exhibits poor temporal resolution; it is susceptible to flash artifacts. PD does not provide velocity nor directional information [139]. The requirement for highly qualified personnel discourages the use of advanced ultrasound units in day-to-day flap monitoring in favor of simpler acoustic solutions.

#### 5.1.2. Implantable Doppler Devices

Implantable Doppler systems are widely used in microsurgery for tissue vitality monitoring, particularly in reconstructive surgeries involving free tissue transfers or flaps [145]. These systems allow for the real-time monitoring of blood flow in critical vessels post-surgery to ensure tissue perfusion, thus detecting potential complications early. At the core of the implantable system is a miniature piezoelectric Doppler probe. This probe is designed to be implanted around a target artery or vein [146]. The ultrasonic transducer converts electrical signals into sound waves and vice versa. The probe is connected to external monitoring equipment via thin flexible wires. These wires are tunneled subcutaneously and remain outside the body for post-surgical monitoring. They provide a link between the implant site and the external Doppler unit. An external Doppler monitor is used to display waveforms that represent blood flow. It also may provide an audible signal indicating flow and patency in the vessel. The electrode is designed to separate from the cuff when a slight tension is applied. In order to avoid accidental disconnection of the probe by pulling on the lead, the lead is connected to an extension cable, which is sutured to the patient and which connects the probe to the monitor [147].

Implantable Doppler probes offer significant advantages in monitoring blood flow in free flap surgeries, particularly in patients with an increased thickness of adipose tissue. Probes’ ability to function independently of flap thickness allows for the effective assessment of blood flow in deeply located vessels. Movement or shifting of the probe can result in a misinterpretation of flow or even false alarms, indicating flow cessation. False positives (indicating flow when there is none) and false negatives (suggesting no flow despite patency) are possible. This can occur due to swelling, hematomas, or misaligned probes [145,148]. The tension exerted on the vessel by the silicone cuff is important, as an excessively tight cuff can cause obstruction to blood flow, while an excessively loose cuff can lead to false-positive results [147]. Implantable Doppler systems are primarily intended for short-term postoperative monitoring, typically within 3–5 days [149]. Long-term implantation is not recommended due to the risk of infection [150]. After reviewing the Manufacturer and User Facility Device Experience data available on the Food and Drug Administration (FDA) website, the authors of the article identified a total of 28 reports related to Cook–Swartz complications. The most frequently reported issue was early detachment of the Cook–Swartz device, which accounted for 13 reports. All cases required reoperation to inspect the flap and place another implantable Doppler. The second most common complication was device malfunction, with eight reports, of which six cases necessitated reoperation. The third most frequent complication involved retained wires, reported in five cases; one patient experienced a subsequent infection that required reoperation to remove the retained filament. The least common complication was failure to disconnect the implantable Doppler, leading to injury to the anastomosed vessels. This occurred in two reports, both of which required reoperation [151].

#### 5.1.3. Flow Coupler

A flow coupler is a specialized device used in tissue vitality monitoring, particularly in microsurgical procedures, such as free flap reconstructions, to ensure adequate blood flow in vessels supplying grafted or transplanted tissue. It integrates anastomotic coupling (connecting blood vessels) with Doppler flow monitoring to provide continuous, real-time feedback on the patency (openness) of blood vessels and tissue perfusion. First, the surgeon uses a ring or clip to join small blood vessels during microsurgery. The materials used for the coupler rings, such as titanium or polymer, are chosen for their biocompatibility, ensuring that they do not trigger an immune response or cause tissue irritation. The rings have small hooks or clips to hold the vessel ends in place, ensuring a tight seal and preventing leakage. The flow coupler integrates a micro-Doppler sensor attached to the coupler ring. The Doppler probe is used to detect blood flow through the anastomosed vessel by emitting and receiving ultrasound waves. The Doppler probes used in flow couplers operate at high frequencies (20 MHz), which is ideal for detecting small-vessel blood flow and microvascular perfusion. The Doppler probe is connected to an external receiver unit via a thin wire. The receiver analyzes the Doppler signal and provides audible feedback. If flow is compromised, the signal changes, alerting the clinician to a potential issue [152].

Like implantable Doppler devices, a major advantage of flow couplers in monitoring individuals with obesity is their capacity to function independently of flap thickness. The device is beneficial when flap capillary refill cannot be monitored or if the flap is entirely buried [153]. The authors of the comparative study emphasize that there are no statistically significant differences in outcomes for free flap breast reconstruction when using either the Cook–Swartz Doppler or the Synovis Flow Coupler to monitor blood flow to the perforator flap (*p* > 0.05). When utilized with proper surgical techniques, both implanted Doppler devices are equally accurate and reliable in detecting potential vascular crises. A key advantage of the Flow Coupler is that it can be used in conjunction with the venous coupler and does not require a separate procedure [154]. It is worth noting that kinking of the vessels by the wire was observed in the literature and required salvage by revision or unkinking of the veins [153,155].

#### 5.1.4. Near-Infrared Spectroscopic Devices

**Figure 2:**
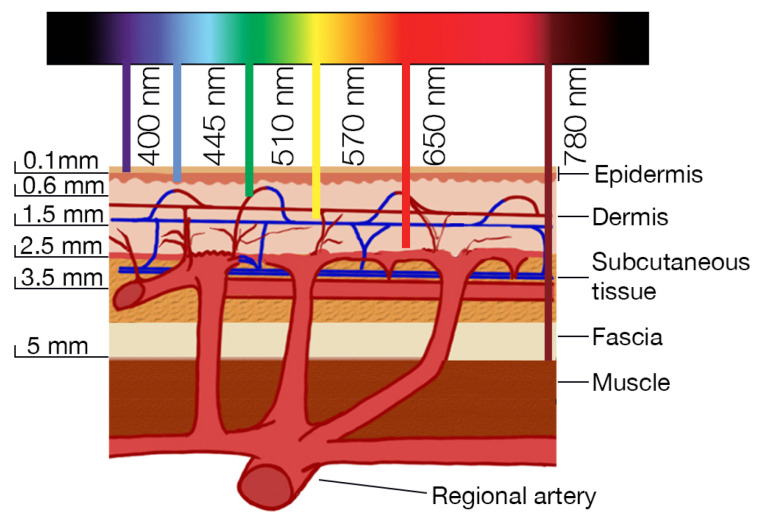
Tissue penetration depth dependent on wavelength of emitted radiation [157].

Near-infrared spectroscopy (NIRS) is a non-invasive technique that uses the near-infrared region of the electromagnetic spectrum to assess the vitality of tissues, primarily through measurements of oxygenation and hemodynamics. NIRS is used to measure changes in the oxygenation levels of tissues, specifically the concentrations of oxyhemoglobin (HbO_2_) and deoxyhemoglobin (HHb). By analyzing these, the total hemoglobin content and the tissue oxygen saturation (StO_2_) can be calculated. NIRS also helps assess blood volume and flow within tissues by tracking changes in the light absorption caused by blood dynamics. This provides insight into the overall health and function of the tissue, including its metabolic status. Typically, systems for tissue vitality use near-infrared light (700–950 nm). The anatomy of the skin and how the perforating branches penetrate its different layers are presented in the Figure 2 conceptual image. The relation between deep and superficial epigastric vessels is pictured here as well. It is noticeable that the superficial vessels lie in the layer of subcutaneous tissue which allows performing no incision in the muscle. The depth of radiation penetration is strictly dependent on the emitted wavelength. This range is chosen because the light in this spectrum can penetrate biological tissues deeply enough. Moreover, the use of two light sources emitting two wavelengths allows for differentiation between oxygenated and deoxygenated hemoglobin [130]. Light-emitting diodes are commonly used for their precise emission at required wavelengths. Probes are designed to measure transmitted or/and reflected light. In terms of tissue flaps, the latter are usually used. Photodiodes or avalanche photodiodes (APDs) are used as detectors to collect the light after it has passed through or reflected from the tissue. One of the greatest advantages of NIRS is an ability to measure blood flow continuously and in real-time, allowing the observation of dynamic changes. In the article [156], researchers demonstrated that PPG monitoring can detect a perfusion abnormality in a very timely fashion—specifically, within 5 min of vascular compromise.

The depth of the penetration varies based on factors like probe placement, tissue type, and patient movement. The method is sensitive to external light; however, advanced NIRS systems can apply noise reduction algorithms and calibration techniques to improve the signal-to-noise ratio. Studies suggest that NIR radiations can penetrate 5–15 mm into the tissue [157,158]. Interestingly, the findings of the study on fat as the propagation medium in optical-based in-body communications provide evidence that the presence of fat layers in porcine samples results in higher received optical power than flesh layers [159]. According to data presented by Kagaya and Miyamoto, the major commercially available devices operate within wavelengths of 475–830 nm. The declared depth of monitoring ability ranges from 2 mm (visible light) up to 24 mm ( near-infrared) [160]. According to Ouyang et al., the detection depth of the third-generation TSAH-100 NIRS device is half the distance between the emitter and detector; therefore, the detection depth of the sensor is 1.5 to 2.0 cm. As a result, it becomes challenging to assess the blood supply of a buried fibular flap when the covering tissue thickness exceeds this limit. This issue is particularly evident in patients with obesity or those experiencing significant postoperative swelling in the area, especially following exploratory surgery [161].

**Figure 3:**
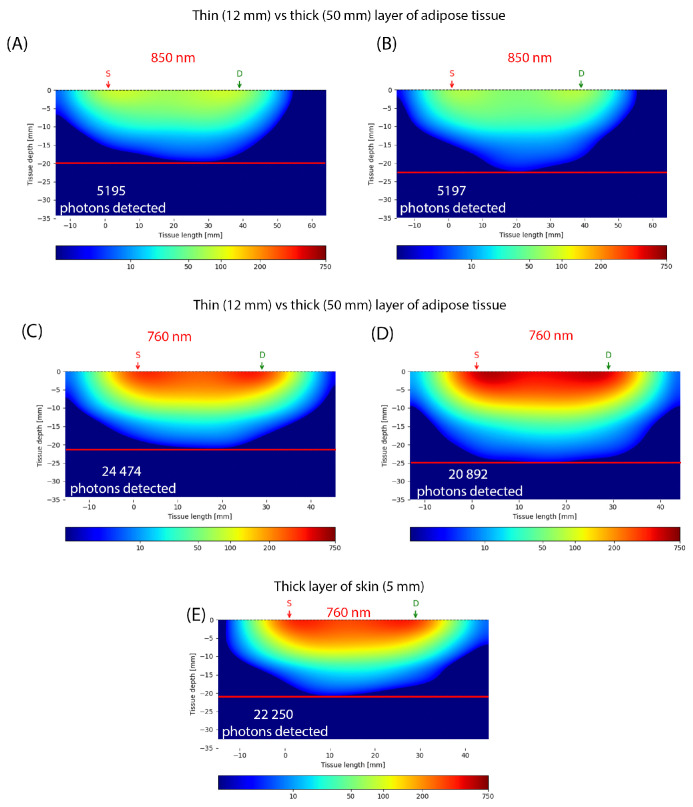
A set of Monte Carlo simulations based on the construction of the sensor modeled after TSAH-100. S—sensor, D—detector, red line—detection depth limit. Simulations have been carried out with the following parameters: (**A**) wavelength: 850 nm, S-D distance: 40 mm, adipose tissue thickness: 12 mm. (**B**) wavelength: 850 nm, S-D distance: 40 mm, adipose tissue thickness: 50 mm. (**C**) wavelength: 760 nm, S-D distance: 40 mm, adipose tissue thickness: 12 mm. (**D**) wavelength: 760 nm, S-D distance: 40 mm, adipose tissue thickness: 50 mm. (**E**) wavelength: 850 nm, S-D distance: 40 mm, adipose tissue thickness: 12 mm, 5 mm layer of skin added [161].

The photon transport in the tissue can be simulated with Monte Carlo methods [162,163]. A set of simulations based on the details provided by Ouyang et al. [161] has been carried out. Each simulation was based on emissions of 10,000,000 virtual `photons’. We simulated ’photon’ behavior in three-layer tissue. The skin thickness was 2.2 mm, the underlying adipose tissue was variable (12 mm and 50 mm), and the underlying muscle had a thickness of 100 mm in order to guarantee no issues with boundaries. The emitter was a point source and the detector measured 5 × 5 mm. For a wavelength of 760 nm, the emitter–detector distance was 3 cm. For a wavelength of 850 nm, the distance was increased to 4 cm. As thickness of the skin can vary depending on the body region [164], a simulation using 760 nm wavelength, with skin thickness increased from 2.2 mm up to 5.0 mm and adipose layer thickness of 12 mm, was conducted. In order to allow for a clear comparison, a power-law normalization function with the gamma parameter set to 0.3 was applied. Based on the observations presented in Figure 3, a closer analysis of the photon distribution shows that thicker adipose tissue allows radiation to penetrate 3–4 mm deeper. However, photons that penetrate the tissue deeper than 10 mm represent only a small fraction of all detected. Moreover, the thickness of the skin hardly influenced the depth of penetration. The observations above suggest that the method should mainly be considered for the observation of superficial vessels. Clinicians should take photon density distribution into consideration while deciding on monitoring perforators with NIRS. In order to locate a perforator of interest, handheld Doppler could be used. It is worth mentioning that according to Kim et al. [165], the Doppler-detected point to actual perforator distance was 12.9 mm.

#### 5.1.5. ICG FA

ICG-FA is a technique used for tissue vitality monitoring, particularly in reconstructive and transplant surgeries. It offers real-time visualization of blood flow and tissue perfusion, aiding surgeons in assessing the viability of tissues or flaps [166]. ICG is a water-soluble, fluorescent dye that is injected intravenously. It binds to plasma proteins and circulates through the bloodstream. It is rapidly cleared by the liver, with minimal side effects. When exposed to near-infrared (NIR) radiation, ICG emits fluorescence, which can be captured by an NIR camera [167]. The tissue penetration depth of near-infrared light-enhanced fluorescence ranges from 0.5 to 1.0 cm [168]. ICG-FA offers real-time feedback on the vascularization of tissues, allowing surgeons to immediately assess blood flow through vessels supplying transplanted or reconstructed tissues. The dynamic assessment of perfusion is possible within seconds of ICG injection. Surgeons can use the fluorescence data for both qualitative visualization and quantitative analysis of tissue perfusion. The color-coded maps can be analyzed to determine the percentage of well-perfused tissue, providing objective data on tissue viability. Although rare, adverse reactions to ICG, such as allergies or anaphylaxis, can occur [169]. Due to the need for dye administration, ICG-FA is unsuitable for continuous or frequent measurement. However, its ability to visualize tissue perfusion makes it a feasible and safe technique for detecting free flap vascular complications [170,171].

### 5.2. Indirect

#### 5.2.1. Laser Doppler Flowmetry

Laser Doppler flowmetry (LDF) is a non-invasive technique used to measure microvascular blood flow in tissues. It relies on the Doppler shift principle. The frequency of the reflected radiation changes proportionally to the velocity of the blood cells [172]. LDF is commonly used to evaluate blood perfusion, monitor skin viability, and assess tissue metabolism. The output of LDF is typically in arbitrary units, representing relative changes in blood flow over time. LDF devices use a low-power laser, typically in the visible or near-infrared range (typically 633 to 810 nm). This wavelength allows deep enough tissue penetration while minimizing absorption by tissue components [173]. The laser radiation is delivered to the tissue through optical fibers. The scattered radiation returning from the tissue is detected by a photodetector, often a photodiode or an avalanche photodiode (APD) [174]. The measurement area is small, typically under 1 mm^2^;. LDF measures flux, which is a combination of both the velocity of red blood cells and their concentration [175]. The output is generally expressed as a relative measure rather than absolute units of flow, as LDF is sensitive to microvascular blood flow rather than large vessel circulation [173]. LDF provides point measurements of blood flow, meaning that the laser beam targets a specific spot on the tissue. To assess larger areas, probes can be moved or multi-point scanning systems can be used. LDF can measure blood flow continuously and in real-time, making it useful for tracking dynamic changes in microcirculation, such as in response to stimuli, temperature changes, or physical activity. A low value of LDF in the examination of a particular patient does not help a doctor in making clinical decisions [176]. Kulikov et al. emphasize the difficulty in distinguishing microcirculatory disorders from those related to hypertension or aging [177]. According to findings by Clough et al. [178], currently, time and frequency descriptors of the LDF signal do not consistently and reliably reflect changes in microcirculation.

#### 5.2.2. Glucose and Lactate Level-Based Devices

Glucose and lactate measurements are key indicators of tissue vitality, providing valuable insights into the metabolic status of tissues. In clinical and research settings, continuous monitoring of these biomarkers helps assess tissue health, energy metabolism, and the balance between aerobic and anaerobic respiration. Most glucose and lactate measurements rely on biosensors, which are devices that detect specific biological molecules (analytes) and convert this detection into a measurable electrical signal. These biosensors detect glucose and lactate through enzyme-catalyzed reactions. Glucose oxidase (GOx) is the most common enzyme used for glucose detection. Glucose oxidase reacts with glucose in the presence of oxygen to produce gluconic acid and hydrogen peroxide (H_2_O_2_). The H_2_O_2_ is then electrochemically oxidized at an electrode, producing a current proportional to the glucose concentration. Lactate oxidase (LO_*x*_) or lactate dehydrogenase (LDH) are used for lactate detection. Lactate oxidase reacts with lactate to produce pyruvate and H_2_O_2_, with a similar electrochemical detection process at the electrode. The working electrode (often platinum or carbon) detects the oxidation of H_2_O_2_. The resulting current is measured and correlated to the concentration of glucose or lactate. Biosensors for glucose and lactate must be calibrated regularly to ensure accurate measurements. Calibration involves exposing the sensor to known concentrations of glucose or lactate and adjusting the system’s output to match these values. Wearable sensors are gaining popularity, especially in sports medicine and fitness tracking. Such solutions are typically implantable [179] or worn on the skin [180]. Glucose and lactate levels might not reflect immediate changes in tissue perfusion. For example, after a sudden vascular occlusion, the metabolic changes leading to lactate accumulation and glucose depletion may take time to manifest. Measurements with handheld devices provide a snapshot of the tissue’s current metabolic state. Short-term assessments can miss critical periods of ischemia or reperfusion injury in tissues. In a study involving measurements of capillary glucose and lactate levels, clinical signs lagged behind the biological manifestations. This delay was 2 h for arterial thrombosis and about 4 h for venous thrombosis [181]. In the study [182], it was demonstrated that needles of 8 mm or longer, when inserted perpendicularly, frequently penetrate muscle in the limbs of males and individuals with a BMI of less than 25 kg/m^2^. For the Continuous Glucose Monitors, the needle length typically varies 5 mm for Freestyle Libre 2, 7.5 mm for Dexcom G6, and 10.5 mm for Guardian [183].

#### 5.2.3. Classical/Static Thermography

Thermography (IR-T) or static thermography (ST) is a non-invasive imaging technique used for tissue vitality monitoring by assessing the skin temperature and underlying perfusion. The core of IR-T is an infrared thermal camera capable of detecting the infrared radiation emitted by the skin. This radiation correlates with the skin’s surface temperature, providing thermal maps of the tissue. High-sensitivity thermal cameras are required to detect subtle temperature differences as changes in blood flow. There are reports of the use of static thermography methods in assessing the blood supply to the subcutaneous tissue in individuals with obesity. Three dominant approaches can be noted: analysis of the temperature distribution of the whole body, analysis of individual body parts, and analysis of the temperature distribution during physical exercise. With the use of infrared thermography, researchers could detect the flap compromise as early as 2 h postoperation in comparison to clinical examination which could detect flap compromise at 6 h at the earliest [184].

#### 5.2.4. Whole-Body Thermographic Assessment

Researchers often use full-body infrared imaging to compare individuals with obesity versus normal weight under both resting and stimulated conditions (e.g., after exercise or a cold/warm stimulus). It is important to remember that research shows that there is no difference in core body temperature between people of normal weight and those who are obese. In [185], it was shown that mean (±SE) daily core body average temperature was not significantly different between the 35 people without obesity and the 46 people with obesity (36.92 ± 0.03 °C compared with 36.89 ± 0.03 °C; *p* = 0.44). Women had a mean core body temperature ≈ 0.23 °C greater than that of men (36.99 ± 0.03 °C compared with 36.76 ± 0.03 °C; *p* < 0.0001). Obese participants tend to show lower average skin temperatures over adipose-rich regions (e.g., abdomen and thighs) compared to lean counterparts [103,104]. Thicker subcutaneous fat acts as an insulator, slowing heat transfer from the body core to the surface. Additionally, reduced local blood perfusion in individuals with obesity can contribute to lower surface temperatures in certain regions [105,106]. In [186], the correlation between the body mass index (BMI) and the skin temperature (Tsk) over the different parts of the body was discussed. The results of this study suggest that individuals who are overweight or have obesity tend to have lower skin temperatures compared to individuals who are underweight or have a healthy weight; e.g., higher differences in Tsk compared to individuals with normal weight were obtained in the anterior trunk, both in the individuals who are overweight (−1.00 °C less) and in the ones with obesity (−2.20 °C; less). This area corresponds to the area with a higher concentration of body fat in males. On the other hand, individuals who were underweight exhibited increased Tsk values for all the considered ROIs, with higher differences in the frontal and rear arms (1.79 °C and 2.16 °C, respectively). Details are presented in the table below (Table 5). The last column contains the value of the observed maximum temperature difference for individual body parts. These values are practically determined among people with varying body weights. It is worth noting that the difference can be up to 3.5 °C and over.

**Figure 4:**
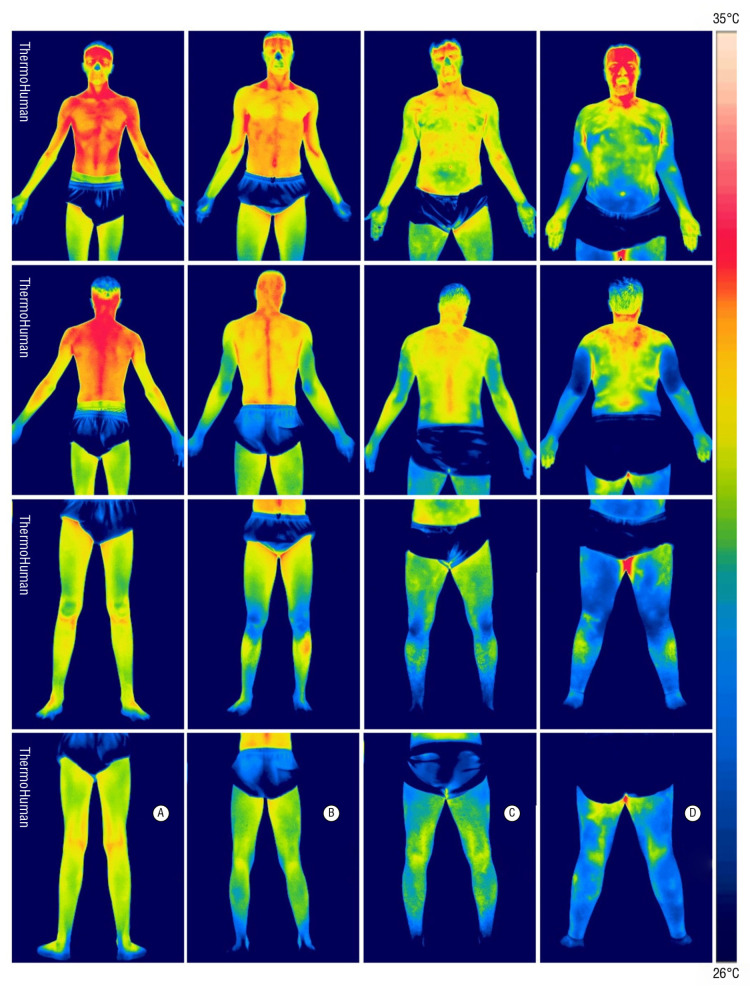
Thermograms of individuals: different body parts (ROIs) for (**A**) slim, (**B**) normal, (**C**) overweight, and (**D**) obese men [186] (with permission from Elsevier).

Figure 4 shows an example of a thermographic examination of four people with different BMIs and body types correlated with the BMI value. In the leftmost column, there is a person with a slim build; in the second column, a person of normal weight; in the third, a person who is overweight; and in the last one, a person with obesity (BMI > 30 kg/m^2^). The examination shows a view from the front and back of the body. The temperature is marked with colors: from blue—the lowest temperature (from 26°)—to red—the highest (up to 36°). It is clearly visible that for a person with obesity, blue predominates, especially on the stomach, arms, and thighs. Similar results were obtained in a study conducted on women with obesity [187]. The highest mean temperature differences were recorded for the abdomen and thigh areas, 2.7° ± 0.4° and 1.8° ± 0.3°, respectively. They also proved that there are no differences in core temperatures between women with a normal body mass index and women who have obesity.

#### 5.2.5. Localized Thermographic Studies (e.g., Hands and Feet)

Infrared thermography (IRT) is also used to assess microcirculatory function in the extremities, which can be altered in obesity and metabolic syndrome. Delayed or attenuated rewarming responses have been observed in individuals with obesity after a cold stimulus (e.g., immersing a hand or foot in cold water), indicating potential vascular dysfunction. Both the insulating effect of subcutaneous fat and possible endothelial impairment can lead to slower temperature recovery in distal regions [103,104,188].

#### 5.2.6. Exercise-Related Thermography

IR-T-based monitoring of skin temperature before, during, and after exercise helps illustrate how obesity influences thermoregulatory responses [106]. Individuals with obesity often exhibit a smaller increase in skin temperature in heavily padded areas (e.g., abdomen and thighs) due to the insulating effects of adipose tissue [104,189]. Conversely, in well-perfused areas with less subcutaneous fat (e.g., the face or hands), the temperature rise can be more pronounced in participants with obesity if local blood flow or sweating mechanisms are differentially affected [106,189].

#### 5.2.7. Active Dynamic Thermography

Active dynamic thermography (ADT) is an extension of well-known static thermography. It requires the application of an external thermal stimulus (e.g., heating or cooling) to the tissue, a thermal imaging camera, and software to analyze transient processes inside the tissue during cooling or heating. This stimulus causes a controlled thermal response in the tissue. Common methods include air jets, contact plates, or laser sources to deliver the thermal stimulus and provoke a temperature change in the tissue. The captured thermal data is processed using specialized software that analyzes the thermal response of the tissue. This software generates a dynamic thermogram, which tracks how quickly the tissue temperature returns to baseline after the stimulus. ADT monitors how the tissue heats up or cools down in response to an applied thermal stimulus. The speed of temperature recovery (referred to as thermal recovery time) correlates with blood flow and tissue perfusion. Well-perfused tissue tends to recover more rapidly due to efficient heat exchange, while poorly perfused or ischemic tissue will exhibit a slower thermal recovery. ADT generates both qualitative thermal images (visual heat maps) and quantitative metrics such as the rate of thermal recovery. This provides objective data that can be analyzed to assess tissue viability. Time-series data allows for a detailed analysis of local tissue metabolism, giving a real-time assessment of blood flow in small and superficial vessels. One of the key technical advantages of ADT is its non-invasive and contactless nature. This reduces the risk of infection or mechanical disturbance to the monitored tissue, which is particularly important in delicate post-surgical scenarios. ADT primarily assesses surface temperatures and is limited in its ability to monitor deep tissue perfusion. It only provides accurate data for superficial layers, meaning that deeper vessels or tissues are not adequately visualized. ADT is highly sensitive to environmental conditions, including ambient temperature and airflow. These external factors can interfere with the thermal measurements and distort the tissue’s thermal recovery profile. Maintaining a controlled environment during the procedure is essential for accurate data collection. High-quality infrared cameras and data processing software used in ADT are expensive. This can make the technology less accessible, particularly in resource-limited settings. ADT offers real-time assessments, but only during the period when the thermal stimulus is applied and the tissue’s response is measured. Long-term continuous monitoring of tissue vitality, like in implantable Doppler systems, clearly hindered with ADT. The interpretation of dynamic thermography results can sometimes be challenging due to non-uniform heat distribution across different tissue types. Variations in skin thickness or underlying fat can affect thermal readings, complicating the assessment of deeper structures.

## 6. Discussion

Each monitoring modality could be influenced by obesity-induced changes in different ways. With an increasing percentage of patients with obesity undergoing reconstructive procedures using tissue flaps, surgeons should choose the appropriate monitoring method based on the patient’s physical characteristics, such as the thickness of the adipose tissue as well as the thickness of the skin in the transferred flap. Not every clinically acclaimed method for perfusion assessment guarantees the successful, timely detection of blood flow impairment in individuals with obesity. Handheld Doppler ultrasound is considered a gold standard by many surgeons—its reliability and ability to observe blood flow even in deep buried vessels are, however, obscured by its noncontinuous nature, causing patients discomfort and requiring frequent attention from qualified medical personnel. The reproducibility of blood flow parameter determination, when using Doppler ultrasound measurements, is parameter-dependent and, in general, poor [190]. The wearable Doppler solutions hold promise because of its ease of use and ability to continuously monitor blood flow [191,192]. Implantable Doppler and flow coupler are currently used for continuous, hands-free blood flow monitoring. Among the biggest advantages, the elasticity in terms of the size of the monitored vessels and the depth at which they are buried should be noted. A considerable downside however is the risk of accidental removal of the probe or even damage of the vessel anastomosis. Hence, the patient’s movements during the monitoring period have to be restricted. Additionally, the constant acoustic signal emitted by the monitoring device can reduce patient comfort; in addition, slight changes in volume associated with reduced blood flow can be easily missed. Given the above, selection of the implantable solution should be considered as a first choice only when alternative solutions should fail. Interestingly, according to the study [148], higher-risk flaps monitored with a Doppler device had a significantly higher return to the operating room rate (21% compared to 4%, *p* < 0.001) and flap failure rate (7% compared to 1%, *p* = 0.002). Salvage rates for free flaps were similar between both groups, at 62% for higher-risk flaps and 60% for lower-risk flaps (*p* = 1.0). Results indicate there is insufficient evidence to suggest that the implantable Doppler reduces the rate of flap failures in routine low-risk cases. Additionally, the effectiveness of using Doppler monitoring for high-risk reconstructions needs to be evaluated in a prospective randomized study. Implantable sensors can cause foreign body reactions or tissue irritation, delaying detection of the tissue ischemia [193]. A similar issue can be observed for the intra-cutaneous sensor for glucose and lactate levels monitoring [194,195]. In addition, such devices provide information on the local metabolic status of the tissue where the sensor or sample is taken. It may not reflect global tissue perfusion or viability, especially in heterogeneous tissue where perfusion might vary significantly across regions. Different tissues (e.g., muscle, fat, skin) have varying baseline levels of glucose and lactate metabolism [196]. This variability makes it difficult to establish standard reference ranges for tissue viability based solely on these biomarkers. Current results [197] indicate that Continuous Glucose Monitor readings for diabetic individuals are more consistent from one day to the next. Inter-day reproducibility was poorest among normoglycemic individuals, especially younger normoglycemic individuals, suggesting the need to monitor some patient groups more often than others. Biosensor-based systems, whether implantable or wearable, can offer real-time, low-invasive monitoring. Combining biomarkers with other monitoring methods, like Doppler ultrasonography, plethysmography, or thermal imaging, can help overcome some of the method’s downfalls and improve outcomes in tissue vitality assessments.

The primary challenge associated with optical-based methods for monitoring patients with obesity is the limited depth of tissue penetration by electromagnetic radiation. Focused light can only penetrate a few millimeters into the body, which means that it primarily allows for the observation of superficial vessels and tissues. This limitation especially applies to LDF, otherwise a valuable tool for real-time, non-invasive monitoring of blood flow. LDF’s effectiveness is diminished by its sensitivity to motion and poor spatial resolution, making it less suitable for assessing deeper tissues or larger areas. According to the recent study [198], laser Doppler probes (number ≥ 4) have a sufficiently high degree of reproducibility after replacing the probes within the same participant. In our clinical experience however, when using LDF, additional verification of underlying vessel patency should be considered. NIRS is considered to be reliable as a postoperative non-invasive flap monitoring tool. It provides real-time insight for monitoring oxygenation and blood flow, crucial for assessing tissue vitality. Postoperative flap monitoring with NIRS was able to detect vascular compromise earlier than other monitoring methods, such as a physical examination [199] or Doppler monitoring [200], and achieved a higher salvage rate. There is evidence in the literature on the level of agreement between NIRS-derived parameters and on the repeatability and reproducibility of selected parameters [201]. In our experience, compared to LDF, NIRS can assess deeper areas of tissue, making it potentially more useful for monitoring patients with obesity. Moreover, current NIRS devices’ performance could be further improved by a differential setup or active noise cancellation circuitry.

ICG-FA provides a real-time assessment of blood flow and tissue perfusion excelling in sensitivity and resolution. However, ICG-FA only provides a snapshot of perfusion at the time of imaging. Continuous, long-term monitoring of tissue viability is not possible with this technique at the moment. ICG-FA systems are relatively expensive, both in terms of the dye used and the specialized NIR camera equipment required. According to the research [202], ICG-FA is a reproducible technique for in vivo evaluation of perforasome perfusion and produces comparable results to classical infrared thermography. Active dynamic thermography allows for the non-invasive and real-time monitoring of tissue vitality, particularly in superficial tissues. Its ability to assess tissue perfusion dynamically using temperature changes offers unique insights into blood flow and tissue health. However, sensitivity to environmental factors, a considerable size of the aperture, and operator dependency must be considered. Despite these challenges, ADT is potentially a very useful technique for the early detection of vascular compromise and ensuring tissue viability in post-surgical care. Given the availability of methods and our experience with thermography, which shows the overall condition of the flap, it still requires significant computational and hardware effort. Static thermography in most cases allows for the detection of temperature differences between regions of interest. In the case of the human body, temperature differences between symmetrical areas, which should be characterized by almost the same temperature, are often analyzed. However, the temperature distribution is often dependent on the ambient conditions: air temperature, humidity, and presence or absence of sources of thermal radiation such as radiators in the room where the study is carried out. Therefore, one should move away from the analysis of the absolute temperature distribution in favor of differential measurements or even in favor of techniques with external excitation ADT and determination of parametric images, such as the distribution of time constants or polynomial coefficients in the TSR (Thermographic Signal Reconstruction) method. A detailed review of excitation techniques and the possibilities of using machine learning methods is included in [203]. It is absolutely necessary to ensure that thermodynamic equilibrium conditions are established during the examination, i.e., the patient’s acclimatization to the given environmental conditions. Before the thermographic examination, the use of stimulants (alcohol, coffee, etc.) should also be eliminated for a specified period of time, as well as various physical activities. Guidelines for preparing for the thermographic examination are included in the American Academy of Thermology Guidelines for Breast screening recommendations [204].

## 7. Conclusions

Participants of the Plastic Surgery 2006 conference, organized by the American Society of Plastic Surgeons, concluded that patients who are severely obese and have undergone a mastectomy should postpone breast reconstruction until they have lost a significant amount of weight [205]. In the more recent literature evidence, obesity increasing the risk of complications in both implant-based and autologous reconstruction can be found [17,206,207,208,209]. Each unit increase in BMI correlates to 7% increased odds of overall complications and 13% increased odds of reconstructive failure [210]. For that reason, currently, at Gdansk Medical University, a BMI over 35 kg/m^2^ is considered a contraindication for reconstructive procedures.

Due to the growing problem of obesity, such patients cannot be disqualified from treatment [206]. Relying solely on the BMI for clinical decisions in obesity treatment is insufficient because it fails to personalize health status assessment. The BMI excludes patients with high muscle mass, as well as patients with gynoid obesity, who have a lower cardiovascular risk. With body mass index limitations taken into account, the Edmonton Obesity Staging System arises as a potential alternative tool for reconstructive procedure qualification. A holistic approach to the patient should allow the mitigation of possible complications of the surgical procedure. Studies suggest that extra patient care should be taken in patients with a body mass index above 35 kg/m^2^ [211].

Parameters of the flap, e.g., the thickness of skin and adipose tissue or the size of the perforators and the depth at which they are located, are influenced by the patient’s weight. When considering the optimal measurement system choice, a patient’s obesity class is not without significance. Monitoring devices should be selected based on established medical knowledge of the conditions. An increase in adipose tissue thickness may cause issues linked to the device’s ability to monitor deeper positioned vessels.

The authors would like to emphasize that the monitoring methods presented in this article should be clinically investigated and compared on a large sample. Based on technical specifications, measurement principles, and results of simulations, some conclusions can be drawn.

Increased fat tissue thickness negatively impacts ultrasound Doppler accuracy by attenuating the ultrasound beam, which causes lower image quality, less penetration, and signal distortion. This can lead to a less clear visualization of blood flow and, in some cases, inaccurate diagnostic interpretations. The distribution of fat is also a key factor, as subcutaneous fat requires deeper penetration than fat located internally [212].

From a technical standpoint, both the implantable Doppler and flow coupler are the most suitable methods for monitoring patients with obesity. The ability to directly verify the quality of anastomosis is invaluable; the device’s performance is independent of adipose tissue thickness and provides direct insight into the blood flow source. A direct observation of perforators with NIRS systems poses significant difficulties. As shown in Monte Carlo simulations, the penetration depth for such systems rarely exceeds 25 mm, with most of the energy concentrated below 10 mm. Deeper vessels may be challenging to observe with this method, especially when considering pulse wave measurements. In patients with obesity, the distance from the tissue surface to the perforator should be established, for example, using ultrasound imaging.

Another approach to assessing tissue vitality is by observing its surface. In such a case, we can seek manifestations of anastomosis failure. Information provided by superficial vessels, such as tissue oxygenation level, has proven useful. Both NIRS and LDF systems can provide such data. The use of biochemical sensors to monitor lactate or glucose levels should also be considered. NIRS, LDF, and biochemical sensors, however, only allow for point and multi-point measurements. Camera-based systems, on the other hand, allow for total flap measurements. ICG-FA provides insight into tissue blood flow, allowing observation of the tissue up to 1 cm. Unfortunately, continuous measurements are impossible—the technique relies on injecting the ICG dye, which is transiently visible. The possibility of frequent measurement could be considered instead. This approach would require solving technical problems such as automated dye injection or real-time image analysis, potentially leveraging artificial intelligence to quantify perfusion metrics rapidly. Another valuable indicator of tissue perfusion is its temperature. In a recent preliminary study, active dynamic thermography was found to be independent of the BMI, indicating that this method may be suitable for patients with obesity [213]. This method, however, poses challenges related to the continuity of measurement. It is important to acknowledge that repetitive tissue cooling can cause discomfort for the patient.

In summary, when selecting measurement techniques for flap monitoring in individuals with obesity, it is beneficial to consider both direct blood flow measurements and superficial measurements. In future considerations, it is important to determine the areas of most significant interest within the flap, aiding the future selection of an adequate technique and spatial resolution needed in specific cases. Additionally, the required resolution and percentage of changes in perfusion should be better characterized and described. Engineers should aim to automate the detection of ischemic areas and trends of changes in relation to the tissues surrounding flaps, consequently reducing the workload on medical personnel and reducing response times.

## Figures and Tables

**Table 1:** National Heart, Lung, and Blood Institute (NHLBI) and World Health Organization (WHO) classification of overweight and obesity [12,13,14].

Classification	BMI (kg/m^2^)	WHO (1997)	WHO (1995)
Underweight	≤18.5	Underweight	–
Normal Range	18.5–24.9	Normal Range	Normal Range
Overweight	25.0–29.9	Preobese	Grade I Obese
Obese I	30.0–34.9	Obese Class I	Grade II Obese
Obese II	35.0–39.9	Obese Class II	
Obese III	≥40.0	Obese Class III	Grade III Obese

**Table 2:** Calculation of percentage body fat from the BMI [15].

Subjects	*n*	Equation	*R* ^2^	Standard Error	Statistical Significance
Men	214	1.402×BMI+0.177×age−22.519	0.52	5.54	<0.001
Women	290	1.592×BMI+0.096×age−11.666	0.56	5.75	<0.001

**Table 3:** Clinical and Functional Stages of Obesity based on [20].

Stage	Description
0	No obesity-related risk factors detected; all health indicators are within normal limits.
1	Obesity-related subclinical risk factors, including borderline hypertension, impaired fasting glucose, and elevated liver enzymes. May present with mild symptoms such as dyspnea during moderate exertion, aches, fatigue, and slight impairment of well-being.
2	Moderate limitations in daily activities and well-being due to obesity-related chronic diseases (e.g., hypertension, type 2 diabetes, sleep apnea).
3	Organ damage (e.g., myocardial infarction, heart failure, diabetic complications, incapacitating osteoarthritis); significant psychopathology, functional limitations, or impairment of well-being.
4	Severe disabilities from obesity-related chronic diseases, disabling psychopathology, major functional limitations, and marked impairment of well-being.

**Table 4:** Summary of studies investigating obesity as a risk factor for postoperative complications in patients undergoing various types of breast reconstruction surgery.

Reference (Year)	*n* (Breasts)	Complications	Average BMI (kg/m^2^)	BMI Range (kg/m^2^)	*p*	Surgery Type	Detection Method	Obesity as a Risk Factor	Country
Seidenstuecker et al. (2011) [29]	79	13	—	≥30	—	DIEP	PE	Yes	Germany
	479	22	—	<30	—	MS TRAM			
Fischer et al. (2013) [32]	812 (1258)	398	28.4	22.8–47.8	—	MS TRAM, DIEP, SIEA	PE	Yes	USA
Knox et al. (2016) [28]	130 (183)	93	25.6 ± 3.5	18–35	0.35	DIEP	PE	—	Canada
	377 (444)	294	25.9 ± 3.8	18.8–43.9	0.35	pTRAM			
Ochoa et al. (2018) [30]	418 (639)	152	28.30	17–42	—	DIEP	Not disclosed	Yes	USA
Wilkins et al. (2018) [31]	2224	263	26.6	21–32.2	<0.001	pTRAM, TRAM, LDF, DIEP, implant	PE	Yes	USA
Palve et al. (2020) [33]	793	380	—	<2 –>30	<0.001	DIEP, LD, TMG, implant	Not disclosed	Yes	Finland
Heidekrueger et al. (2021) [34]	3911	293	—	<18.5–34.9	<0.001	DIEP	Not disclosed	Yes	Germany
Yoshino et al. (2021) [35]	129	67	23.72 ± 3.66	20.2–27.38	<0.001	DIEP	Not disclosed	Yes	Japan
Barnes et al. (2024) [38]	218 (313)	71	—	18.96–30.00	<0.001	DIEP, SIEA	Not disclosed	Yes	USA
	147 (232)	76	—	30.01–57.68	<0.001	MS TRAM, VRAM			

*n*—number of cases; *p*—statistical significance of BMI impact on complications; DIEP—Deep Inferior Epigastric Perforator; TRAM—Transverse Rectus Abdominus Muscle; LD—Latissimus Dorsi; pTRAM—Pedicled TRAM; MS TRAM—Muscle Sparing TRAM; SIEA—superficial inferior epigastric artery; VRAM—vertical rectus abdominis muscle; TMG—Transverse Myocutaneus Gracilis; PE—physical examination.

**Table 5:** Skin temperature ($T_{sk}$) in different body regions of interest (ROIs) across various weight categories [186].

Integrated ROI	Mean $T_{\mathrm{sk}}$ (±SD, °C)				$\Delta T_{\mathrm{sk}}$ (°C)
	Underweight	Healthy Weight	Overweight	Obesity	Max–Min
Frontal view					
Arms	33.14 ± 0.55	31.35 ± 0.63	30.77 ± 0.54	30.45 ± 0.75	2.69
Trunk	34.20 ± 0.39	32.90 ± 0.43	31.90 ± 0.56	30.70 ± 0.81	3.50
Legs	33.00 ± 0.82	31.28 ± 0.82	30.84 ± 0.74	30.71 ± 0.95	2.29
Rear view					
Arms	32.51 ± 0.62	30.35 ± 0.73	29.81 ± 0.65	28.98 ± 0.75	3.53
Trunk	30.41 ± 0.28	29.39 ± 0.33	28.74 ± 0.56	28.05 ± 0.77	2.36
Legs	31.60 ± 0.61	30.60 ± 0.74	29.20 ± 0.71	29.10 ± 0.69	2.50

## Data Availability

No new data were created or analyzed in this study. Data sharing is not applicable to this article.
